# The Influence of Hospitalization and Nursing Interventions on Inpatients' Sleep Quality: A Multicenter Observational Study

**DOI:** 10.1111/wvn.70162

**Published:** 2026-08-30

**Authors:** Filip Bellon, Adriano Targa, Isabel Perez‐Loza, Eva Abad‐Corpa, Esther Rubinat‐Arnaldo, María Isabel Orts‐Cortés, Montserrat Gea‐Sánchez

**Affiliations:** ^1^ GESEC Group, Department of Nursing and Physiotherapy, Faculty of Nursing and Physiotherapy University of Lleida Lleida Spain; ^2^ Biomedical Research Institute of Lleida – Dr. Pifarré Foundation, IRBLleida Lleida Spain; ^3^ Translational Research in Respiratory Medicine, Biomedical Research Institute of Lleida (IRBLleida) Hospital Universitari Arnau de Vilanova‐Santa Maria Lleida Spain; ^4^ CIBER of Respiratory Diseases (CIBERES) Institute of Health Carlos III Madrid Spain; ^5^ Critical Care Cardiology Unit, IDIVAL Nursing Research Group, Venia Docendi UC Marqués de Valdecilla University Hospital Santander Spain; ^6^ University of Murcia‐Murcia Health Service (IMIB‐Arrixaca) Murcia Spain; ^7^ Institute of Health Carlos III Nursing and Healthcare Research Unit (Investén‐isciii) Madrid Spain; ^8^ Institute of Health Carlos III Biomedical Research Center for Fragility and Healthy Aging (CIBERFES) Madrid Spain; ^9^ Center for Biomedical Research on Diabetes and Associated Metabolic Diseases Instituto de Salud Carlos III Barcelona Spain; ^10^ Nursing Department, Alicante Institute for Health and Biomedical Research (ISABIAL, Group 23) University of Alicante (BALMIS) Alicante Spain

**Keywords:** hospitalization, nursing, sleep, sleep deprivation

## Abstract

**Background:**

Sleep quality is a critical component of health and recovery, yet it remains a significant challenge during hospitalization. Poor sleep among inpatients is common and worsens throughout hospital stays, adversely affecting health outcomes and patient wellbeing. Despite its importance, sleep often receives limited attention within hospital policies and nursing practices.

**Aim:**

To evaluate the impact of hospitalization and night‐time nursing interventions on the sleep quality of noncritical patients in Spanish public hospitals.

**Methods:**

An observational, multicenter study was conducted across 12 public hospitals. Data were collected from patients admitted to surgical and medical units between January 2020 and February 2022. Sleep quality was assessed using the Pittsburgh Sleep Quality Index at hospital admission and discharge. Night‐time nursing interventions (including medication administration, vital signs monitoring, blood sample collection, and anxiety management interventions), sociodemographic, anthropometric, clinical variables, and self‐reported disturbances were also recorded. Statistical analyses included descriptive statistics and linear regression models adjusted for potential confounding factors.

**Results:**

The study included 383 patients. Most patients (71%) presented poor sleep quality upon admission, which worsened during hospitalization (85.9%). A significant association was found between the number of intervention days and poorer sleep quality. Collection of blood samples was significantly associated with worsened sleep quality in female patients, while anxiety management was associated with lower sleep quality in males. Discomfort with roommates and night‐time nursing interventions were also related to worse sleep quality.

**Linking Evidence to Action:**

Hospitalization exacerbates poor sleep quality in noncritical patients. Night‐time nursing interventions, discomfort with the hospital environment, and anxiety management are key factors. Interventions addressing these modifiable factors could improve patients' sleep quality and recovery. Hospital policies should incorporate sleep as a key quality indicator, promoting strategies to reduce unnecessary night‐time interventions and environmental disturbances. Nursing education must emphasize sleep‐friendly care practices and appropriate planning of night‐time activities.

## 1 Background

Sleep quality is regarded as a complex clinical concept and includes both qualitative and quantitative characteristics, such as the number of awakenings that occur during the night, along with feeling refreshed and rested upon waking versus feeling tired at waking and throughout the following day (Buysse et al. 1989). Sleep is essential to human health, contributing to the daily functioning and quality of life (Ramar et al. 2021). Human beings spend about one third of their lives sleeping; as a result, it is the activity that takes up the most of our time (Biddle and Hamermesh 1990). Nevertheless, previous research demonstrated that sleep problems are common in Europe, with a prevalence in adults that ranges from 16% to 31% (van de Straat and Bracke 2015), Notably, insomnia, identified as one of the most common sleep disorders, has an estimated prevalence of approximately 12.4% (van Straten et al. 2025).

Sleep is a relevant process for health and quality of life, and alterations in this regard are associated with a plethora of adverse outcomes. These negative effects can prejudice various organ systems (Chang et al. 2020), and may lead to higher risk of multiple complications such as delirium (Pisani and D'Ambrosio 2020), hyperglycaemia and impaired fasting glucose, or cardiovascular disease (Liu and Chen 2019), among others. Decreased sleep quality also diminishes individuals' pain thresholds by elevating the levels of neurotransmitters associated with pain sensitivity (Li et al. 2022), which can lead to higher levels of anxiety, depressive symptoms, and diminished mood (Weinrib et al. 2017).

The importance of sleep in hospital care has been emphasized throughout history and can be considered a major component of health care. Accordingly, Florence Nightingale argued this in the late 1800s, describing that a patient should never be waked intentionally (Nightingale 1992). Given the restoring and healing properties of sleep, good sleep quality is particularly important for hospitalized patients (Kryeger et al. 2021). However, several studies report poor sleep quality during hospitalization (Altman et al. 2017; Delaney et al. 2018; Jakobsen et al. 2020). Moreover, Adams et al. (2024) observed that sleep disturbances are common in hospital, and this can persist up to 12 months after hospital discharge (Altman et al. 2017).

Several factors including the environment, the health condition, and the psychological state of the patients can affect their sleep quality during hospitalization. The hospital environment poses significant challenges to sleep quality, primarily stemming from misaligned artificial lighting, limited exposure to natural sunlight, nocturnal disturbances caused by excessive noise, and atypical meal timings (Honarmand et al. 2020; Wesselius et al. 2018). In addition, hospitalized patients need constant care, which leads to frequent interruptions during the night‐time period (Fąfara et al. 2018). Accordingly, previous studies demonstrate that one of the most common causes of sleep disruptions in hospitals is noise, followed by night‐time nursing care interventions (Adams et al. 2024; Wesselius et al. 2018). However, such studies are mainly focused on critically ill patients, a particular context in terms of environmental noise, continuous lighting, and regular monitoring (Ritmala‐Castren et al. 2015).

## 2 Methods

### 2.1 Aim

We aimed to evaluate the impact of hospitalization, its context, and night‐time nursing interventions on the sleep quality of noncritical patients. To our knowledge, this is the first multicenter study addressing this matter in Spain. The findings of this study will offer important insights in factors that disturb inpatients' sleep.

### 2.2 Study Design

This observational, multicente study was conducted to gather data from patients admitted to surgical and medical units. The study protocol was registered with Clinicaltrials.gov (NCT04113876). This study adhered to the Strengthening the Reporting of Observational Studies in Epidemiology (STROBE) guidelines.

### 2.3 Participants and Setting

Patients from 12 hospitals across Spain were recruited between January 2020 and February 2022. All hospitals belonged to the Spanish National Health System and covered both rural and urban areas. The inclusion criteria comprised: (i) being hospitalized in surgical or medical units, (ii) aged 18 years or older, and (iii) consenting to participate in the study voluntarily. The exclusion criteria comprised patients who suffered from visual or auditory impairment, intellectual disability, or cognitive impairment as documented in their medical record.

### 2.4 Data Collection

Data were collected on sociodemographic and anthropometric variables, clinical characteristics of patients, hospital‐related information, nursing night‐time care interventions, self‐reported disturbance factors, and sleep quality.

#### 2.4.1 Sociodemographic, Anthropometric, and Clinical Variables

We collected sociodemographic and anthropometric data (sex, age, body mass index [BMI]), educational level, employment status, and habits such as smoking and intake of recreational drugs. Clinical characteristics such as comorbidities and intake of sleep‐related medication, and hospitalization‐related information such as reasons of admission were reviewed within the medical record of each included patient and compiled by the nursing staff on the respective ward. Additionally, nurses were asked to report on room type (single or shared) and length of hospital stay after hospital discharge.

#### 2.4.2 Nursing Care Interventions

Nightshift nurses were asked to record the number and type of night‐time nursing care interventions performed between 12 p.m. and 06 a.m. through a diary during the first 4 days of hospitalization. A total of 31 types of nursing interventions that could potentially disrupt patients' sleep were selected from the Nursing Interventions Classification (NIC) (Butcher et al. 2018).

#### 2.4.3 Self‐Reported Disturbances

Patients were requested to rate, on a 10‐point Likert‐type scale, the disruption caused by their roommate (if applicable) and night‐time nursing activities. Furthermore, patients were asked if they experienced discomfort with the bed, room temperature, and bedding employing a dichotomous question with options “yes” or “no.”

#### 2.4.4 Sleep Quality

Patients' sleep quality was evaluated through the Pittsburgh Sleep Quality Index (PSQI). The PSQI is composed of several questions representing one of the seven components of sleep quality: subjective sleep quality, sleep latency, sleep duration, sleep efficiency, sleep disturbance, sleep medication intake, and daytime dysfunction. Each component score is rated on a 3‐point scale, leading to a sum of up to 21 points. Higher scores indicate worse sleep quality. A score greater than 5 indicates poor sleep quality, whereas a PSQI score less than or equal to 5 indicates good sleep quality (Buysse et al. 1989). We used the validated Spanish version of the PSQI (Hita‐Contreras et al. 2014). The assessments were performed at the hospital admission referring to the patients' sleep quality during the previous month, and at hospital discharge referring to the patients' sleep quality during the hospital stay.

### 2.5 Statistical Analysis

Absolute and relative frequencies were used for qualitative data. For quantitative variables, central tendency and dispersion measures were used depending on whether their distribution could be considered normal (mean and standard deviation) or not (median and interquartile range) according to the Shapiro–Wilk test. Given the long list of comorbidities and pharmacological treatments obtained from clinical histories, a variable clustering analysis using Hoeffding's *D* statistic as a similarity measure was performed to reduce the number of them to adjust for. Missing values were imputed by applying regression and classification recursive partitioning trees for quantitative and qualitative variables with missing information, respectively. These trees were pruned according to the minimum cross‐validated error before being used for imputation. Next, we used an ordinary least squares multivariable regression to model the patient‐reported sleep quality at hospital discharge and estimate its adjusted association with each of the following variables of interest: hospital stay duration (days), number of intervention days, number of different interventions, type of intervention (introduced by indicator variables), room type, disturbance level caused by room partner/s, by interventions, by bed, by temperature, and by bedding. The adjustment variables for every regression model included the patient‐reported sleep quality at hospital admission, sex, age, BMI, the reduced list of comorbidities, and pharmacological treatments. Finally, we performed a unique ordinary least squares regression model with all the variables of interest but with only one of the variables related with interventions, specifically, the intervention variable reaching the highest coefficient of determination. All models were also stratified by sex. All statistical analyses were performed with R Statistical Software and a significance level of 0.05 was applied.

## 3 Results

### 3.1 Baseline Characteristics

Our sample was composed of 383 patients (58.18% males), with a median [IQR] age of 64.0 [55.0; 74.0] years old, and a median [IQR] BMI of 26.6 [23.4; 30.1] kg/m^2^. The most prevalent comorbidities were cardiovascular diseases (48.8%), hypertension (44.1%), endocrine diseases (31.9%), and digestive diseases (30.0%).

Patients spent a median [IQR] number of 8.0 [7.0; 11.0] days at the hospital for reasons such as digestive diseases (26.4%), neoplasms (23.1%), and respiratory diseases (19.1%). The vast majority of the patients (79.5%) stayed in shared double rooms.

The most frequent night‐time nursing care interventions performed were medication administration (78.9%), vital signs control (54.0%), pain management (38.4%), collection of blood samples (36.3%), and management and reduction of anxiety (23.0%). More detailed information about baseline characteristics of the cohort is reflected in Table 1.

**TABLE 1:** Baseline characteristics of the cohort.

	Global, *n* = 383
Sociodemographic and anthropometric data	
Sex, male	222 (58.1%)
Age (years)	64.0 [55.0; 74.0]
BMI (kg/m^2^)	26.6 [23.4; 30.1]
Educational level	
Primary education	200 (53.8%)
Secondary education	127 (34.1%)
Higher education	45 (12.1%)
Employment status	
Working	109 (28.83%)
Unemployed	39 (10.3%)
Retired	226 (59.8%)
Studying	4 (1.06%)
Habits	
Tobacco	82 (21.5%)
Recreational drugs	13 (3.43%)
Comorbidities	
Cardiovascular diseases	187 (48.8%)
Hypertension	169 (44.1%)
Endocrine diseases	122 (31.9%)
Digestive diseases	115 (30.0%)
Hospitalization	
Duration (days)	8.00 [7.00–11.00]
Room type, double	303 (79.5%)
Type of nursing intervention	
Medication administration	302 (78.9%)
Vital sign control	207 (54.0%)
Pain management	147 (38.4%)
Collection of blood samples	139 (36.3%)
Anxiety management	88 (23.0%)
Reasons for hospitalization	
Digestive diseases	101 (26.4%)
Neoplasms	89 (23.1%)
Respiratory diseases	73 (19.1%)
Cardiovascular diseases	61 (15.9%)
Musculoskeletal diseases	38 (9.9%)
Sleep‐related medication	
Psychotropics	41 (10.7%)
Opiates	66 (17.2%)
Hypnotics‐sedatives	127 (33.2%)

*Note:* Qualitative data are represented as *n* (%). The medians [p_25_; p_75_] were estimated for quantitative variables. Missing data: education level: 11; employment status: 5; tobacco: 1; recreational drugs: 4; hospital stay: 46.

Abbreviations: BMI, body mass index; *n*, number; p, percentile.

### 3.2 Sleep Quality

**FIGURE 1:**
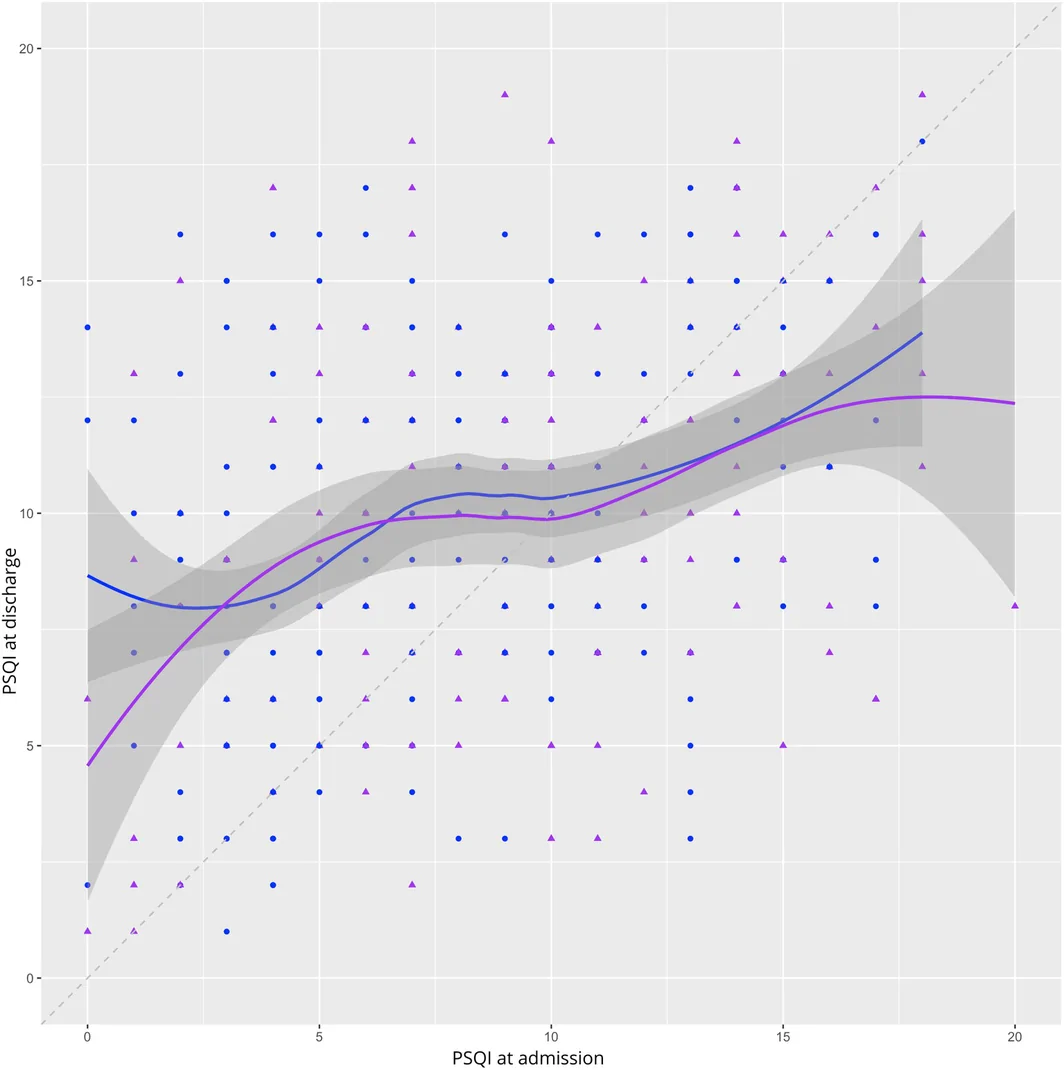
Smoothed relationship between pre‐hospitalization and during‐hospitalization Pittsburgh Sleep Quality Index scores for the overall sample and stratified by sex.

According to the PSQI, most patients (71%) presented poor sleep quality upon admission, with a median [IQR] score of 9 [5.0; 12.0]. This prevalence increased during hospitalization with 85.9% of patients presenting poor sleep quality with a median [IQR] score of 10 [7.0: 13.0], leading to a median change (95% CI) of 1.5 [1.0; 2.0] (*p* < 0.001) points between both time‐points (Figure 1).

Analysis stratified by sex revealed that the median change in the PSQI score between time‐points was 2 [1.5; 3.0] (*p* < 0.001) among males and 0.5 [0.0; 1.5] (*p* = 0.176) among female patients.

The prevalence of patients based on the changes in good and poor sleep quality measured by the PSQI scores from before to during hospitalization is presented in Supporting Information File 1.

### 3.3 Hospitalization‐Related Factors, Night‐Time Nursing Interventions, and Sleep Quality

PSQI scores of hospitalized patients were examined in relation to several objective aspects associated with hospitalization and nursing night‐time interventions through the use of linear regression models adjusted for potential confounding factors. Results for the overall sample and subgroups based on sex are detailed in Tables 2 and 3.

**TABLE 2:** Sleep quality (PSQI) according to objective hospitalization‐related information.

	Global		Male		Female	
	Estimates (95% CI)	*p*	Estimates (95% CI)	*p*	Estimates (95% CI)	*p*
Duration	−0.03 (−0.07–0.00)	0.073	−0.03 (−0.08–0.02)	0.240	−0.03 (−0.09–0.02)	0.244
Room type (shared room)	0.14 (−0.75–1.04)	0.751	0.42 (−0.67–1.51)	0.455	−0.46 (−2.15–1.24)	0.595
Number of intervention days	0.10 (0.05–0.15)	**< 0.001**	0.11 (0.05–0.17)	**< 0.001**	0.07 (−0.02–0.16)	0.103
Number of different interventions	0.32 (0.18–0.45)	**< 0.001**	0.30 (0.13–0.47)	**< 0.001**	0.31 (0.06–0.57)	**0.017**

*Note:* Each estimate of change in PSQI for each variable was obtained from an independent linear regression model adjusted by baseline characteristics including assessing the sleep quality by PSQI according to objective hospitalization‐related information. The models were adjusted by pre‐hospitalization PSQI, sex, age, BMI, clinical history (specifically, previous diagnostics of neoplasia, immune system diseases, endocrine diseases, mental disorders, neurological disorders, hypertension, respiratory diseases, digestive diseases, skin diseases, diseases of the musculoskeletal system, diseases of the genitourinary system, and infectious diseases), patient‐reported comorbidities (specifically, hypothyroidism, restless legs or sleep apnoea) and pharmacological treatments (specifically, hypnotics, hormonal treatment, psychotropics, anticoagulants, and opiates). Bold values indicate statistically significant results (*p* < 0.005).

**TABLE 3:** Sleep quality (PSQI) according to the main carried out nursing night‐time interventions.

Type of intervention	Global		Male		Female	
	Estimates *β* (95% CI)	*p*	Estimates *β* (95% CI)	*p*	Estimates *β* (95% CI)	*p*
Medication administration	0.88 (−0.08–1.84)	0.072	0.94 (−0.25–2.13)	0.119	0.84 (−0.96–2.64)	0.356
Vital sign control	0.05 (−0.78–0.87)	0.910	0.45 (−0.59–1.50)	0.387	−0.75 (−2.19–0.69)	0.304
Acute pain management	0.33 (−0.48–1.15)	0.419	0.06 (−1.05–1.16)	0.919	1.10 (−0.26–2.47)	0.111
Chronic pain management	−0.35 (−1.91–1.22)	0.664	−1.27 (−3.94–1.41)	0.351	0.59 (−1.69–2.86)	0.610
Collection of blood samples	0.36 (−0.41–1.12)	0.362	−0.04 (−1.03–0.95)	0.939	1.44 (0.03–2.85)	**0.045**
Anxiety management	1.35 (0.34–2.37)	**0.009**	2.06 (0.68–3.45)	**0.004**	0.12 (−1.50–1.75)	0.880

*Note:* Each estimate of change in PSQI for the type of nursing intervention was obtained from a linear regression model including indicator variables (one per intervention) representing their performance during the first 4 days of hospitalization and adjusted by baseline characteristics including assessing the sleep quality (PSQI) according to objective hospitalization‐related information. The models were adjusted by pre‐hospitalization PSQI, sex, age, BMI, clinical history (specifically, previous diagnostics of neoplasia, immune system diseases, endocrine diseases, mental disorders, neurological disorders, hypertension, respiratory diseases, digestive diseases, skin diseases, diseases of the musculoskeletal system, diseases of the genitourinary system, and infectious diseases), patient‐reported comorbidities (specifically, hypothyroidism, restless legs, or sleep apnoea), and pharmacological treatments (specifically, hypnotics, hormonal treatment, psychotropics, anticoagulants, and opiates). Bold values indicate statistically significant results (*p* < 0.005).

Significant associations between the number of days with intervention and the global PSQI score were observed (*β* = 0.10 [0.05, 0.15], *p* < 0.001) indicating that more intervention days were associated with poorer sleep quality (Table 2). This was consistent for male patients (*β* = 0.11 [0.05, 0.17], *p* < 0.001), but not significant for female patients (*β* = 0.07 [−0.02, 0.16], *p* = 0.103). In addition, the number of different interventions received during the hospital stay was associated with a significant increase in the global PSQI score (*β* = 0.32 [0.18, 0.45], *p* < 0.001), which was similar between male and female patients. No associations between the PSQI score and factors such as duration of hospitalization and room type were observed.

Among the most frequently performed night‐time nursing interventions, the collection of blood samples was significantly associated with worsened sleep quality in female patients (*β* = 1.44 [0.03, 2.85], *p* = 0.045); however, this factor was not significant for male patients. Receiving anxiety management consistently indicated lower sleep quality, with significant associations observed in the global sample (*β* = 1.35 [0.34, 2.37], *p* = 0.009), and in males (*β* = 2.06 [0.68, 3.45], *p* = 0.004). Other specific night‐time interventions were not found to be significantly associated with sleep quality in our sample (Table 3).

### 3.4 Self‐Reported Disturbances and Sleep Quality

Patients rated on a 10‐point‐Likert‐type scale the discomfort with night‐time nursing care interventions, which revealed a median of 3.00 [1.00, 6.00] out of 10, and discomfort with roommates were scored with a median of 2.00 [0.00, 6.00]. A total of 125 patients (32.6%) reported that room temperature interfered with their sleep, 116 (30.3%) patients' sleep was affected by poor bed comfort, and 63 (16.4%) indicated to have suffered sleep disturbances because of the bedding.

Linear regression models adjusted for confounding factors revealed that discomfort due to roommates was significantly associated with higher PSQI score in the global sample (*β* = 0.24 [0.14, 0.35], *p* < 0.001). Stratified analyses confirmed this finding for both males (*β* = 0.19 [0.05, 0.33], *p* = 0.007) and females (*β* = 0.31 [0.12, 0.49], *p* = 0.001). Additionally, discomfort with nursing night‐time interventions was related to worse sleep quality in the global sample (*β* = 0.37 [0.25, 0.49], *p* < 0.001), with males (*β* = 0.40 [0.23, 0.56], *p* < 0.001) experiencing a slightly stronger effect compared to females (*β* = 0.29 [0.07, 0.51], *p* = 0.011).

We also observed an increase of 1.28 points (0.50, 2.06, *p* = 0.001) in the PSQI score among patients reporting discomfort due to bed conditions, with a significant effect for both male and female patients. Patients reporting discomfort due to bedding were significantly associated with a 1.50 (0.54, 2.46, *p* = 0.002) increase in the PSQI global score. Stratified analysis revealed that this effect was significant only among female patients (*β* = 1.65 [0.12, 3.17], *p* = 0.034). Temperature discomfort had no significant effect on the sleep quality of our sample (Table 4).

**TABLE 4:** Sleep quality (PSQI) according to subjective hospitalization‐related information.

	Global		Male		Female	
	Estimates (95% CI)	*p*	Estimates (95% CI)	*p*	Estimates (95% CI)	*p*
Discomfort due to roommate	0.24 (0.14–0.35)	**< 0.001**	0.19 (0.05–0.33)	**< 0.007**	0.31 (0.12–0.49)	**0.001**
Discomfort due to nursing interventions	0.37 (0.25–0.49)	**< 0.001**	0.40 (0.23–0.56)	**< 0.001**	0.29 (0.07–0.51)	**0.011**
Discomfort due to bed	1.28 (0.50–2.06)	**0.001**	1.09 (0.05–2.13)	**0.040**	1.33 (0.06–2.60)	**0.041**
Discomfort due to temperature	0.72 (−0.04–1.47)	0.063	0.99 (−0.02–2.01)	0.056	0.41 (−0.79–1.62)	0.499
Discomfort due to bedding	1.50 (0.54–2.46)	**0.002**	1.20 (−0.14–2.54)	0.080	1.65 (0.12–3.17)	**0.034**

*Note:* Each estimate of change in PSQI for each variable was obtained from an independent linear regression model adjusted by baseline characteristics including assessing the sleep quality by PSQI according to objective hospitalization‐related information. The models were adjusted by pre‐hospitalization PSQI, sex, age, BMI, clinical history (specifically, previous diagnostics of neoplasia, immune system diseases, endocrine diseases, mental disorders, neurological disorders, hypertension, respiratory diseases, digestive diseases, skin diseases, diseases of the musculoskeletal system, diseases of the genitourinary system, and infectious diseases), patient‐reported comorbidities (specifically, hypothyroidism, restless legs, or sleep apnoea), and pharmacological treatments (specifically, hypnotics, hormonal treatment, psychotropics, anticoagulants, and opiates). Bold values indicate statistically significant results (*p* < 0.005).

## 4 Discussion

The aim of this study was to evaluate the impact of hospitalization and its context on the sleep quality of noncritical patients. Our findings revealed poor sleep quality in a great percentage of participants at the time of admission. Still, such percentage increased with hospitalization. Possible factors associated with this outcome were the number of days with intervention, number of different night‐time nursing care interventions, and the type of intervention, particularly those associated with anxiety management. Furthermore, self‐reported disturbances such as discomfort with the night‐time nursing care interventions, with the roommate, with the hospital bed, and with the bedding were closely associated with higher scores in the PSQI.

The present findings corroborate those previously reported (Kulpatcharapong et al. 2020; Matsuda et al. 2017), which demonstrated a poor sleep quality in up to 91% of hospitalized patients of different surgical and medical units. Our cohort presented poor sleep quality percentages very similar to these studies. This can be partially explained by the period of recruitment. Accordingly, the study took place during the COVID‐19 pandemic, a context related to alterations in the circadian rhythms and to a compromised sleep quality (Targa et al. 2021).

Moreover, we observed that patients who needed anxiety management‐related interventions tended to sleep worse during their hospital stay. This finding aligns with previous research indicating an increase in anxiety levels and sleep difficulties as side effects of the COVID‐19 pandemic (Jahrami et al. 2021). However, this could also suggest a disconnect between current care processes and the specific needs of anxious patients, highlighting a crucial opportunity to improve hospital and nursing care (Kassahun et al. 2022). Traditional anxiety management strategies such as the provision of information or the use of pharmacological interventions may not be sufficient or adequate in the current context, suggesting the need to adapt nursing interventions to better address these difficulties, considering the impact of external events such as hospitalization. The results indicate a correlation between higher PSQI scores and the anxiety management intervention. However, this relationship may be confounded by the anxiety itself, which is known to contribute to poor sleep quality rather than being directly attributable to the type of intervention (Chellappa and Aeschbach 2022).

Although our analysis showed that poor sleep quality was already common at admission and further deteriorated during hospitalization, our examination of the associated factors provides additional insight into how sleep is considered in routine inpatient care. Several variables linked to poorer sleep can suggest that sleep is not yet consistently integrated into the organization of nocturnal care activities. The subtle consequences of poor sleep quality in the short‐term does not aid in this context. Accordingly, the impact of poor sleep quality is more visible and pronounced in the long term, particularly in sustained conditions that go beyond the average length of hospital stay. This interpretation aligns with previous studies demonstrating that nurses often overestimate the sleep quality of their patients, and frequently identify different factors as possible disturbances (Delaney et al. 2018). Furthermore, a significantly variability exists in how to assess sleep quality, which can further complicate evaluations (Matsuda et al. 2017). The improvement of the sleep quality in hospitalized patients has the potential to positively effect, both on short‐ and long‐term patients' comfort, safety, and health outcomes (Hillman 2021). Considering this, integrating sleep more systematically into routine clinical decision‐making, supported by a valid and reliable instrument to assess a patients' sleep (Gellerstedt et al. 2020), may represent a relevant opportunity for improving the quality of inpatients care.

Nurses have a primary role in ensuring patient care during sleep. Dreher (1996) highlighted the importance of sleep, stating that “destructive activities that impair sleep should be of concern to any nurse.” However, this is challenging in several ways. Environmental disturbing factors are often difficult to modify in the context of public hospitals; each patient has its own sleep habits, and operational models in hospitals are organization‐focused, which makes the provision of patient‐centred care very difficult for nurses despite their best intentions. Likewise, the night‐time nursing care itself has a high impact on the quality of sleep. Our findings demonstrated that an increased number of intervention days and a greater variety of interventions provided to patients during the night were significantly associated with poorer sleep quality. The nursing interventions identified in our study as the most frequent during the night are largely likely to be modified, either by reducing their frequency, by organizing nursing care that permits their patients a night of undisturbed sleep, or by implementation of sleep‐friendly technology. A practical example: the clinical rationale for the collection of blood samples carried out in the early morning is unclear. Sorita et al. (2014) provided evidence of quality improvement interventions altering the timing of routine blood draws from early morning to midnight, demonstrating a more favorable response from patients, a better distribution of workload, and an improvement in the efficiency of care. Another example is vital sign control. Night shift nurses often experience a dilemma between the control of vital signs during the night and the sleep of the patient. In fact, there exists difficulty in applying early warning score algorithms and unclear guidelines for the ideal frequency of vital sign checks (Hope et al. 2018). Given the relevance of vital sign control in the prevention of major events like death or cardiac arrest, its avoidance is extremely dangerous. However, prior research suggests promising results for the screening of vital signs by use of infrared thermography, RGB‐thermal image sensors, bed‐based contactless mattress sensors, or a patient‐worn monitor (McDuff 2023; Selvaraju et al. 2022).

In this study, no differences in PSQI global score were found for room type. However, the vast majority (79.5%) of the patients stayed in shared rooms, which might explain these results. Additionally, our data showed a statistically significant increase in discomfort with nursing interventions and roommates, which could indicate that single rooms provide patients with better sleeping conditions, as they could experience disturbances from roommates and interventions carried out on roommates. The connection between longer duration of sleep and single rooms was shown in previous research (Dobing et al. 2016). Also, patients experiencing disturbance with environmental factors such as bed comfort and bedding had a negative association with their sleep quality. These results are in line with other studies detecting these factors as disturbing for hospitalized patients (Chauny et al. 2019; Morse and Bender 2019).

Several previous studies (Chung et al. 2018; Morse and Bender 2019) showed promising results on the implementation of strategies to prevent the development of sleep–wake disorders by quiet time protocols or educational programs for nursing staff to minimize night time disturbances in order to improve the sleep quality of hospitalized patients. However, more research is needed to confirm the effectiveness of these interventions.

### 4.1 Limitations

Our findings should be interpreted considering some aspects. First, we did not perform an objective evaluation of sleep. While the PSQI is a validated and highly reliable questionnaire, the outcomes could be affected by the subjective nature of the patients' responses. Second, the PSQI was designed to investigate the sleep quality considering the 30 days previous to the time of evaluation. Patients with a hospitalization duration of less than 30 days were advised that the answers at the discharge should be based exclusively on their sleep quality during the hospital stay. Still, this might have led to a subestimation of sleep given that it was not possible to answer “less than 1 week” in some parts of the questionnaire. Third, the recruitment of patients and data collection occurred during the COVID‐19 pandemic, an unprecedented and particular context, with restrictions in terms of mobility, family visits, but also other factors may also be considered. For example, healthcare workers, and especially nurses, have been under extreme pressure, which may have affected the quality and interaction of care. In addition, changes in treatment protocols and isolation measures may have increased patients' stress and feelings of loneliness. Together, these factors may have influenced patients' emotional and physical well‐being, potentially affecting sleep‐related outcomes.

## 5 Implications for Practice and Future Research

Our findings reveal a high prevalence of patients from Spanish public hospitals experiencing poor sleep quality upon admission, a condition exacerbated by hospitalization. This outcome may be linked to various factors, including the number of night‐time nursing care interventions and other disturbances such as discomfort with roommates, hospital beds, and bedding. Given the modifiable nature of these factors, implementing interventions to address them is feasible and holds promise for enhancing sleep quality, thereby improving patients' quality of life and aiding in their recovery.

## 6 Linking Evidence to Action

- *Prioritize sleep quality in hospitals*: Recognize poor sleep quality as a prevalent issue in noncritical patients, with implications for recovery and overall health. Implement protocols to enhance sleep quality from admission to discharge.
- *Modification of night‐time nursing interventions*: Reduce the frequency and variety of night‐time nursing interventions that disrupt sleep to improve patient sleep quality.
- *Improve hospital environment*: Address discomfort related to roommates, hospital beds, and bedding. Consider strategies such as room assignments and environmental adjustments to minimize sleep disturbances.
- *Educate and train nursing staff*: Provide education on the importance of sleep and training in sleep‐friendly practices, promoting awareness and implementation of strategies to minimize night‐time disruptions.
- *Explore technological solutions*: Investigate and adopt sleep‐friendly technologies and methods, such as contactless monitoring systems, to minimize disturbances associated with essential night‐time care activities.

## Funding

This work was supported by Carlos III Institute of Health, PI18/00732, PI18/00743, PI18CIII/0012, CP23/00095.

## Ethics Statement

The relevant fieldwork permission was obtained with the approval of the study by the ethical committees of all participating hospitals, along with the ethical committee of the Carlos III Health Institute (HIP CI CEI PI 182019‐V3).

## Conflicts of Interest

The authors declare no conflicts of interest.

## Supporting information


**Supporting Information: File 1.** Prevalence of changes in good and poor sleep quality.

## Data Availability

The data that support the findings of this study are available from the corresponding author upon reasonable request.
