# Supplementary material for: The Influence of Hospitalization and Nursing Interventions on Inpatients' Sleep Quality: A Multicenter Observational Study
Source: Worldviews Evid Based Nurs. 2026 Aug 30;23(4):e70162. doi: 10.1111/wvn.70162 (PMC13526904; doi:10.1111/wvn.70162)
Supplement: Supplementary file 1 [file WVN-23-0-s001.docx]

Supplementary file 1: prevalence of changes in good and poor sleep quality

| Changes in good (≤5) and poor (>5) sleep quality before and during hospitalization | | |
| --- | --- | --- |
| PSQI before | **PSQI during** | **N (%)** |
| <=5 | <=5 | 33 (8.6%) |
| >5 | <=5 | 21 (5.5%) |
| <=5 | >5 | 78 (20.4%) |
| >5 | >5 | 251 (65.5%) |
| Changes in the classification of the PSQI, with McNemar test, p-value < 0.0001 | | |
